# Case report: Localized xanthogranulomatous pyelonephritis in children: A case report and literature review

**DOI:** 10.3389/fped.2022.952989

**Published:** 2022-07-18

**Authors:** Qi-Fei Deng, Han Chu, Bo Peng, Xiang Liu, Yong-Sheng Cao

**Affiliations:** The Second Department of Pediatric Urology Surgery, Anhui Provincial Children's Hospital, Children's Hospital of Fudan University-Anhui Campus, Hefei, China

**Keywords:** xanthogranulomatous pyelonephritis, diagnosis, treatment, pediatric, partial nephrectomy

## Abstract

**Background:**

Xanthogranulomatous pyelonephritis (XGPN), which is featured by inflammatory destruction of renal parenchyma and fibrosis of kidney, occurs mainly among adults, sporadically among children and rarely among infants. Recurrent urinary tract infections, kidney stone-induced obstructive nephropathy, malnutrition, abnormal lipid metabolism, hypoimmunity, lymphatic obstruction and congenital urinary abnormalities may all cause XGPN among children. Its primary treatment is radical nephrectomy.

**Case description:**

In this study, we describe a rare case of XGPN in a 7-year-old boy infected with Staphylococcus aureus (S. aureus). The child presented with symptoms including recurrent fever, urine culture negative. The postoperative pathology confirmed XGPN. Besides, partial nephrectomy was performed.

**Conclusion:**

XGPN, as a special type of chronic pyelonephritis, is a rare pyelonephritis requiring surgical treatment. Early diagnosis and treatment are crucial to reducing its morbidity and mortality. Although radical nephrectomy is the primary therapeutic option for patients with XGPN, partial nephrectomy surgery should be considered for focal XGPN, aiming to preserve residual renal function in children as far as possible.

## Introduction

Xanthogranulomatous pyelonephritis (XGPN) is a rare and severe chronic granulomatous inflammatory disease of the kidney characterized by substantial infiltration of inflammatory cells and granulomas into the renal parenchyma (1). Its prevalence is low among children, while relatively high among adults. To the present, the number of child cases is less than 300 (2). The etiology of XGPN remains unclear, and it is mostly believed that the urinary flow obstruction and chronic bacterial infection are associated with the development of XGPN (3). The clinical manifestations of XGPN are non-specific, including unexplained fever, abdominal pain, weight loss, anemia or palpable renal mass (4). In this study, we describe a case of XGPN in a child with *S. aureus* infection who underwent kidney-sparing surgery.

## Clinical case

**Figure 1:**
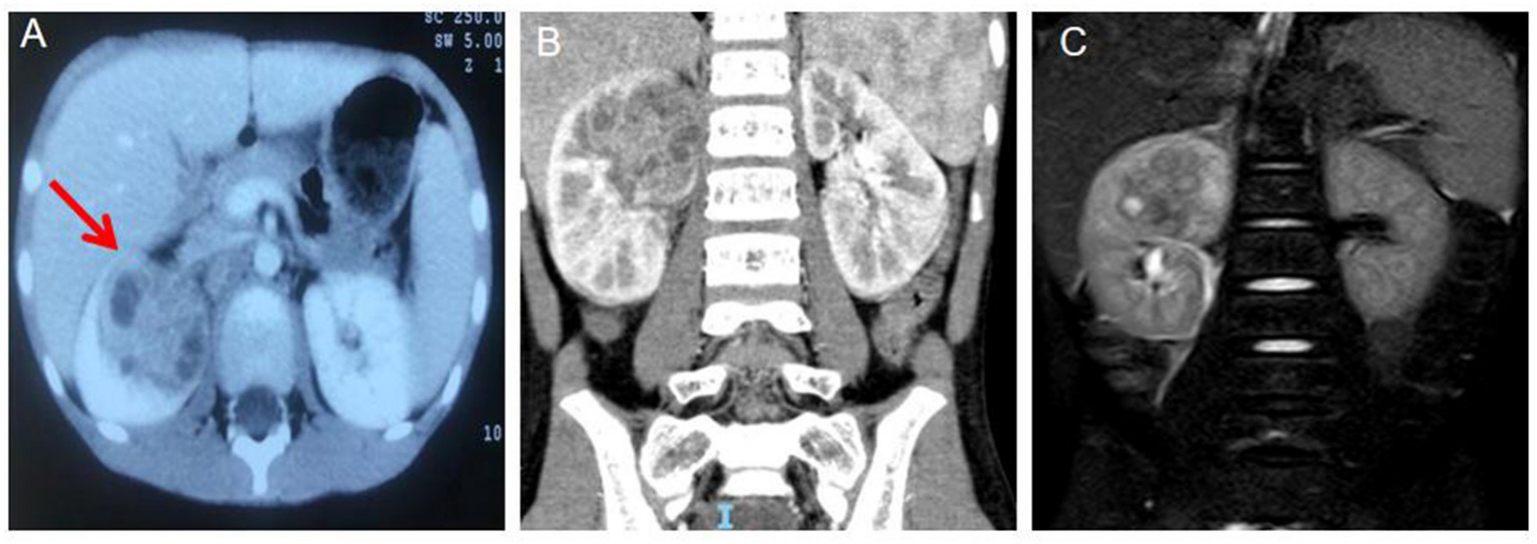
**(A)** Computed tomograph (CT) Arterial period abnormal density shadows were seen in the middle and upper poles of the right kidney, with flaky low-density areas inside and annular enhancement at the edges (the red arrow). **(B)** Coronal view the abnormal density shadow in the upper pole of the right kidney. **(C)** Magnetic resonance (MRI) mass in the upper pole of the right kidney with inhomogeneous signal, unclear boundary, scattered lacunae, delayed inhomogeneous enhancement after enhancement, and thickening of perirenal fascia.

A 7-years-old boy was admitted to the Infectious Department of our Hospital on June 21 of 2021 due to “repeated fever for over half a month”. Half a month ago, the child had repeated fever without obvious inducement, whose body temperature was up to 39 °C. Before the fever, he did not have symptoms like chills, convulsions, runny nose cough, rash, skin infection, vomiting, diarrhea or joint pain. Ultrasound at a local hospital suggested a right renal cystic mass. The child received anti-infective therapy for half a month at a local hospital, which yielded undesirable control of body temperature. During the disease course, the child' weight drop by 1–1.5 kg, good mental state, defecation once weekly and normal urination. Post-admission routine examination: 2021.06.22 WBC 14.51^*^10^∧^9/l; NEUT 68.80%; CRP 57.7 mg/l; ESR 86 mm/h; PCT 0.091 ng/ml; IL-6 54.630 pg/ml; blood and urine cultures (-); mycobacterium tuberculosis antibody (-); liver + kidney functions, electrolytes and urine routine found no obvious abnormalities, and five tumor biomarkers were normal. On 2021.07.05 WBC 13.72^*^10^∧^9/l; NEUT 69.2%; CRP 36.81 mg/l; ESR 81 mm/h; PCT 0.153 ng/ml; IL-6 45.46 pg/ml. Urinary ultrasound on admission revealed a heterogeneous echo mass (about 5.5 × 4.3 × 4.2 cm in size) at the upper pole of the right kidney extending to the middle, with several scattered flaky liquefactions inside it, and the lesions showed point–strip blood flow signals. The lower half of the kidney was normal, the right perirenal fascia was swollen, while the left kidney showed no abnormalities. In terms of imaging examination, contrast-enhanced CT revealed heterogeneous density of the upper pole of the right kidney, with slightly enlarged shape (Figures 1A,B). The renal parenchymal density enhancement was less intense after enhancement, and scattered low-density foci and peripheral annular enhancement were observed. No hydronephrosis or ureteral dilatation was noted. MRI: The mass in the upper pole of the right kidney had heterogeneous signal and unclear boundary, with scattered lacunae inside it (Figure 1C). The heterogeneous enhancement was delayed after enhancement, and the perirenal fascia was thickened. On 2021.07.02 Urinary ultrasound examination revealed that the upper poles of the right kidney were heterogeneous and had slightly hyperechoic areas (infection plus partial liquefaction was considered). The acoustic transmission inside the hyperechoic areas was poor, and there was no good puncture zone.

## Treatment process

**Figure 2:**
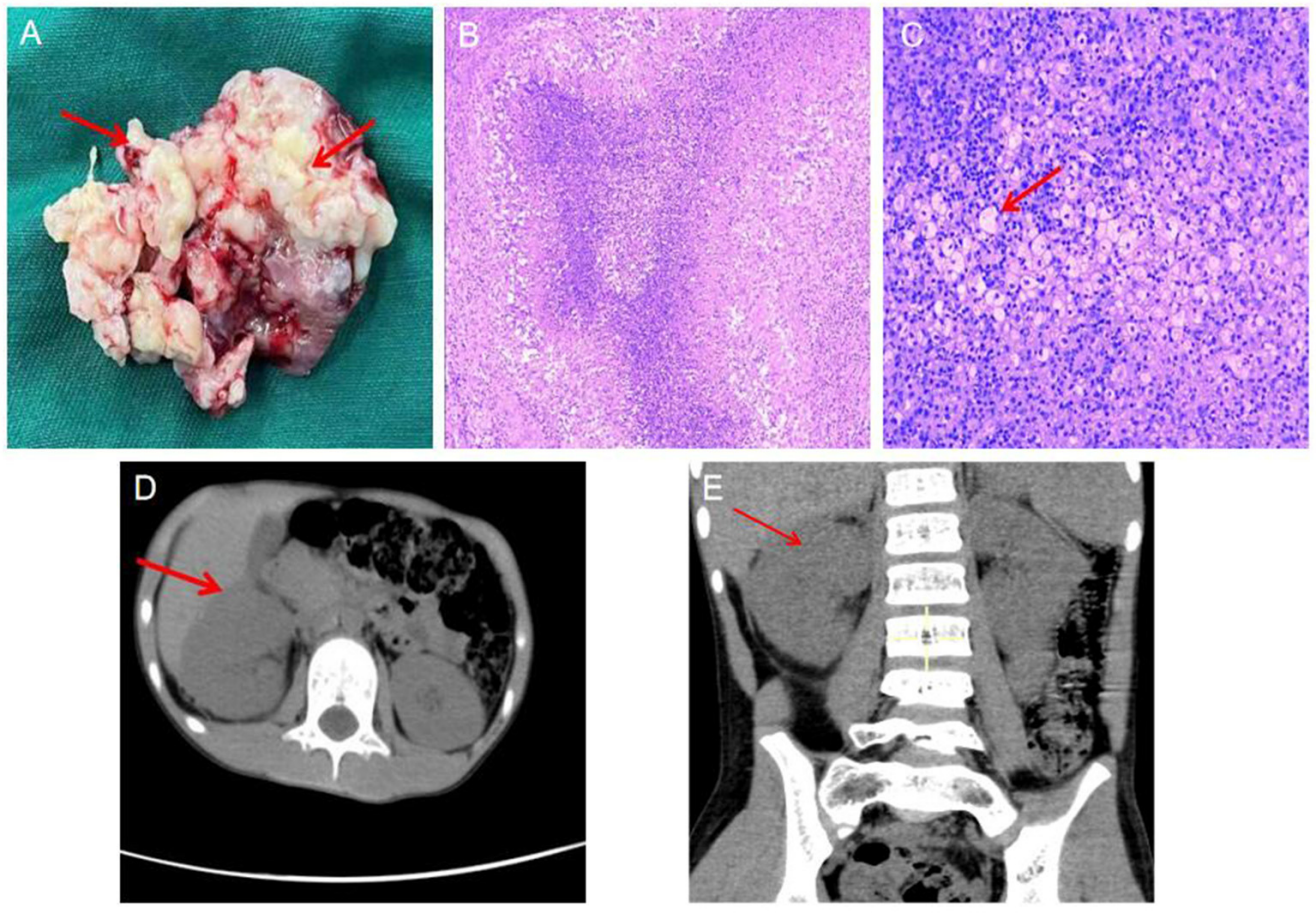
**(A)** Lesion tissue specimen: Multiple central necrotic granulomas in the medulla of renal tissue can be seen, and pus can be seen in the necrotic area, (the red arrow:pus); **(B)** Histopathological examination, low magnification (HE, 5×) abscess formation in the renal parenchyma, with fibrin in the center Necrotic tissue, surrounded by foam cells, fibrous tissue hyperplasia, and surrounding. **(C)** High magnification (HE, 100×), a large number of foam cells reacted around the abscess necrosis. (the red arrow:foam cell). Postoperative follow-up CT showed good preservation of renal structure and function. **(D)** axial image; **(E)** coronal image, (the red arrow: postoperative renal).

After being treated wtih anbitiotics at a lower level hospital for half a month, the child was admitted to the hospital infectious department, and received anti-infective therapy sequentially with ceftazidime (2021.06.21–06.23), imipenem and cilastatin (2021.06.24–06.28), vancomycin (2021.06.28–07.05) and fosfomycin sodium (2021.06.28–07.05). His body temperature did not drop pronouncedly, the fever spikes was maintained at 39°C. The anti-infective therapy yielded poor efficacy, the diagnosis was unclear. The “cystic-like lesion at the upper pole of right kidney” was considered, puncture and pus extraction were impossible, and space-occupying lesions could not be completely ruled out. After multidisciplinary (imaging, oncology, pathology, urology and infection) consultation, the child was transferred to the department of pediatric urology surgery on 2022.07.05 for surgical resection of the lesion and intraoperative frozen pathology. The decision between retention and removal of the kidney was made according to the frozen pathology results. On 2021.07.07, the child underwent right partial nephrectomy under general anesthesia. Intraoperatively, a space-occupying lesion about 5 × 5 × 4 cm in size was observed at the upper parts of the right kidney, the capsule was intact, and the boundary with surrounding normal renal tissue was good (Figure 2A). The mass and perirenal fat were completely enucleated for intraoperative frozen pathology, which revealed no abnormality in the residual renal tissue and presence of inflammatory changes. Therefore kidney retention was decided. Vancomycin was given postoperatively to fight infection, and his body temperature returned to normal 3d postoperatively. Intraoperative pus specimen culture: *S. aureus*. Postoperative pathology results: XGPN (Figures 2B,C). The child was in stable condition 12 d postoperatively, who was thus discharged.

Imaging examinations were performed separately at 1 month and half a year postoperatively, which revealed no recurrence of renal lesions, and good structure and functionality of the retained kidneys (Figures 2D,E).

## Discussion

XGPN is an extremely rare and severe chronic pyelonephritis, with only 200~ child cases to date. The report of XGPN was first launched by Schlag-enhaufer in 1916, and it was not until 1944 that Osterlind started to use this term (5). The etiologies of XGPN are diverse, such as recurrent urinary tract infections (*Escherichia coli, Proteus mirabilis* and rarely *Pseudomonas*), kidney stone-induced obstructive nephropathy, malnutrition, abnormal lipid metabolism, altered immune responses and lymphatic obstruction. Congenital urinary abnormalities have been reported to predispose individuals to this rare infection of the renal parenchyma (6). Admission to the hospital for medical history, defecation once weekly, CT showed residual rectal stool. The child was diagnosed with constipation, and there was an increased risk of recurrent urinary tract infection. No urinary calculi, congenital malformations of urinary system and previous history of urinary tract infection were found. Hence, this disease was not considered, where further exploration was needed.

The clinical manifestations of XGPN are non-specific and diverse, including abdominal pain, weight loss, fever, anemia, symptoms of lower urinary tract infection, abdominal mass and hematuria (7). The study of Stoica I et al. on 66 children with XGPN found that the most common clinical symptoms were low back pain, systemic disease, fever and urinary tract infection, and these non-specific symptoms were present in over half of the patients (8). Based on a study by Al-Ghazo et al., XGPN was associated mostly with the urinary tract obstruction and bacterial infection. Its early clinical symptoms included accelerated ESR, urinary tract infection and anemia. Where the infection spread, symptoms including psoas abscess, parapelvic cysts, renal atrophy and renal rupture would occur (9). Therefore, early diagnosis and treatment are beneficial to protecting the kidney functionality. The patient in the present case only had recurrent fever in the early stage, whose clinical symptom was not obviously specific.

Such abnormal laboratory findings of XGP have been reported: leukocytosis, thrombocytosis, anemia, elevated inflammatory markers (ESR, CRP), and positive urine cultures (10, 11). According to relevant studies, for over half of the patients (both adults and children) with positive urine cultures, the major bacteria in urine cultures were *Proteus vulgaris* and *Escherichia coli*, with the difference being presence of Gram-positive bacteria in urine cultures (12, 13). For the patient in the present case, both the preoperative blood and urine cultures were negative. The pus specimen was collected only during the surgery, and *S. aureus* was cultured, showing inconsistency with the previously reported common bacteria, which was rare. We speculate that *Staphylococcus aureus* spread to the kidneys through hematogenous. It is possible that in a S. aureus infection, the bacteria spread to the kidneys, where they colonize. When the child with low immunity, bacterial load increases and persistent chronic infection leads to a series of abnormalities in the kidneys, until finally the formation of xanthogranulomatous pyelonephritis. Ultrasound and contrast-enhanced CT are greatly vital in the diagnosis of XGPN. According to the ultrasound and imaging findings, the size and structural changes of the diseased kidney and the perirenal invasion and contralateral kidney status could be identified, and the disease could be categorized into either diffuse or focal type (14). Depending on the range of lesion involvement, the XGPN is divided into three stages: grade I (intrarenal type); grade II (perirenal type); and grade III (extrarenal type) (15). The lesions in the present patient were confined primarily to the middle and upper renal poles, while protruded partially from the renal capsule, along with thickening of the perirenal fat sacs. The XGPN in the child reported herein should be of grade II focal type.

Considering the rarity of pediatric XGPN and our lack of relevant knowledge, the clinical manifestations and imaging signs of XGPN are non-specific, and misdiagnosis is highly likely since the differential diagnosis includes a large group of diseases, containing Wilms tumor, renal cell carcinoma, renal abscess, renal tuberculosis, malakoplakia and transitional renal cell carcinoma (16). Studies have reported that adult patients with XGPN are easily misdiagnosed as renal cell carcinoma or renal tuberculosis, while children with XGPN are easily misdiagnosed as renal tumors, with focal XGPN more likely to be misdiagnosed as the renal cell carcinoma, and diffuse XGPN more likely to be misdiagnosed as the renal tuberculosis. Pathological diagnosis is the precise standard for XGPN, and corresponding pathological features include the foam cells formed by substantial macrophage infiltration inside renal parenchyma and the multiple granulomas composed of various inflammatory components (e.g., neutral granulocytes, lymphocytes, plasma cells, cholesterol clefts and multinucleated giant cells), of which the foam cells are specific diagnostic indicators (17). Since the XGPN has low incidence and its clinical and imaging manifestations lack specificity, its diagnosis rate is low. In addition to, the disease is often detected in the late stage, with a high rate of nephrectomy.

The therapeutic measures of XGPN are classified into the conservative and surgical treatments. Conservative treatments are primarily antibiotic therapies. It is recommended to select antibiotics based on the susceptibility tests. Extended-spectrum penicillins (e.g., piperacillin-tazobactam), cephalosporins (ceftriaxone or cefotaxime) and ampicillin + gentamicin are all appropriate therapeutic options. Considering the safety of pediatric medication, cephalosporins and extended-spectrum penicillins are generally used. A study carried out Kim et al. found that for children with mild XGPN, antibiotic therapy could achieve full rehabilitation. Conservative treatment usually lasts for approximately 2 weeks. If the high fever persists and the condition shows insignificant improvement, surgery should be considered (18). Depending on the disease type, antibiotics + partial nephrectomy are generally adopted for focal XGPN, while nephrectomy is generally adopted for diffuse XGPN. Partial nephrectomy is curative in localized forms of XPGN. Due to the extensive perinephric inflammatory adhesions, excision of the perirenal soft tissues is of paramount importance. Nonetheless, XGPN is mostly discovered in the late stage, for which nephrectomy is the primary surgical procedure, in accordance with multiple retrospective studies (8, 9). Xie et al. found that 28~ days of preoperative antibiotics could reduce postoperative complications prominently (19). Thus, early diagnosis and treatment are particularly crucial to protecting the renal function of children. For the child patient in the present case, the contrast-enhanced CT revealed ring enhancement around the upper pole mass of the kidney, so tumor could not be excluded. He had persistent fever, and the nature of the mass in upper renal pole could not be determined. Due to the poor efficacy of anti-infective therapy, surgical exploration was adopted for completely removing the lesions in the upper renal pole. After excluding malignancy, the normal kidney was preserved.

Although postoperative pathology showed a good prognosis, the risk of urinary tract infection, hypertension, renal amyloidosis was still increased. Long-term follow-up should be performed after surgery, especially in the pediatric population.

## Conclusion

As a rare chronic inflammatory disease, XGPN seldom occurs among children and is easily misdiagnosed. Its preoperative diagnosis remains a big challenge, especially in the case of focal type. Although the clinical diagnosis can be assisted by the increasingly accurate ultrasound B and CT, the final diagnosis needs to rely on histopathology. Antibiotic therapy and nephrectomy are the mainstays of treatment for XGPN, but among pediatric patients, partial nephrectomy remains practical. Individualized therapeutic regimens are crucial, especially for children.

## Data availability statement

The raw data supporting the conclusions of this article will be made available by the authors, without undue reservation.

## Ethics statement

Written informed consent was obtained from the individual(s), and minor(s)' legal guardian/next of kin, for the publication of any potentially identifiable images or data included in this article.

## Author contributions

Y-SC and Q-FD: conception and design. HC: collection and assembly of data. XL and BP: data analysis and interpretation. All authors writing the manuscript and final approval of manuscript.

## Funding

This study was supported by Anhui Provincial Health Commission. The funding body played no role in the design of the study and collection, analysis, and interpretation of data and in writing the manuscript.

## Conflict of interest

The authors declare that the research was conducted in the absence of any commercial or financial relationships that could be construed as a potential conflict of interest.

## Publisher's note

All claims expressed in this article are solely those of the authors and do not necessarily represent those of their affiliated organizations, or those of the publisher, the editors and the reviewers. Any product that may be evaluated in this article, or claim that may be made by its manufacturer, is not guaranteed or endorsed by the publisher.
